# Pesticides and public health: discussing risks in Brazilian agro-industrial growth

**DOI:** 10.3389/ftox.2025.1442801

**Published:** 2025-03-13

**Authors:** Juliana E. Perobelli

**Affiliations:** Laboratory of Experimental Toxicology, Marine Institute, Universidade Federal de São Paulo, UNIFESP, Santos, São Paulo, Brazil

**Keywords:** agrochemicals, one health, experimental toxicology, brazilian agriculture, male reproduction, endocrine disruptors

## Abstract

The benefits of pesticides in enhancing agricultural yields are widely accepted by the general public. However, it is essential to address the limitations of the current agricultural model to develop more sustainable practices that prioritize environmental and human health. Brazil, a major global agricultural player, ranks among the top five agro-food producers and exporters, making it one of the largest consumers of pesticides worldwide. Notably, approximately 30% of pesticides used in Brazil are banned in the European Union. Paradoxically, some of these banned agrochemicals re-enter Northern markets through imported agro-food products. Addressing the regulatory disparities between Northern and Southern countries necessitates global initiatives and research to better understand the real biological risks associated with pesticide exposure, particularly concerning reproductive health, endocrine disruption, and carcinogenesis—key targets of these chemicals. Since 2001, the Brazilian Health Regulatory Agency (ANVISA) has operated the “Reports on Pesticide Residue Analysis in Food (RPRAF)” program to evaluate pesticide residues in food samples collected across Brazil. Despite its limitations, the program has been crucial in identifying the chemical exposome related to Brazilian agro-foods, facilitating studies on relevant pesticides, their doses, routes, and exposure schedules, and enabling the development of pre-clinical studies based on real-life exposure scenarios. A thorough understanding of the main mechanism of toxicity is crucial for raising awareness about the health risks associated with pesticide exposure, fostering tailored health strategies and guiding informed regulatory policies.

## 1 Introduction

Pesticides encompass a broad spectrum of chemicals derived from both synthetic and natural sources, employed to control damaging pests across agriculture, forestry, to control disease vectors like mosquitoes, and land management. These substances function by preventing, destroying, repelling, attracting, or mitigating the impact of pests, weeds, and microorganisms (Alavanja, 2009). Pesticides can be classified in various ways, including by target organisms (e.g., insecticides, herbicides, fungicides, rodenticides), chemical structure (e.g., carbamates, organophosphates, organochlorines), mode of action (e.g., acetylcholinesterase inhibitors, GABA-gated chloride channel blockers, sodium channel modulators, juvenile hormone receptor modulators), or by application timing and method (e.g., contact pesticides, foliar pesticides, preplant, pre-emergent, or post-emergent herbicides) (NIPHM, 2025). The majority of pesticides produced are utilized for agricultural purposes, accounting for approximately 85% of the total Brazilian market (Cassou, 2018).

The widespread use of pesticides began in earnest following World War II (1939–1945) due to the urgent need to boost food production after the war’s devastating effects on agriculture (Bernardes et al., 2015). Since then, pesticide use has consistently increased, becoming a cornerstone of modern agriculture and a highly profitable global market. Remarkably, since 2018, just four companies controlled 70% of the global pesticide market, i. e., Bayer AG - Germany, Syngenta AG - Switzerland (which was acquired by China National Chemical Corporation ChemChina), Corteva Agriscience - United States, and BASF SE - Germany (Abrasco, 2024). Economic and industrial forecasts predict that the global agrochemical market will reach approximately $280 billion by 2030 (Statista, 2024; MarketsandMarkets, 2024).

Although the benefits of pesticides in enhancing agricultural yields are widely acknowledged, it is crucial to consider some studies that indicate that only a small fraction, estimated at around 1% of the total pesticide application, actually targets the intended pests. The majority of these chemicals dissipate into the environment, affecting non-target organisms and ecosystems (Bernardes et al., 2015; Tudi et al., 2021). Simultaneously with this, the Food and Agriculture Organization of the United Nations (FAO) reports that approximately 40% of global agricultural production is still lost to pests (FAO, 2022). This significant loss, despite the intense pesticide use, highlights some concerns about the effectiveness of current pesticide practices and modern agricultural methods, revealing the significant environmental and health costs associated with them. The widespread use of pesticides can boost the development of pest resistance, resulting in even greater pesticide use. This triggers a complex array of problems, including harm to non-target organisms, compromising biodiversity, soil degradation, and water pollution, disrupting ecosystems and, consequently, impacting crops health and creating a cycle of unsustainable practices.

## 2 Brazil in the global scenario

### 2.1 Why Brazil Matters

Currently, Brazil stands out as a relevant player in global agriculture, ranking among the top five agro-food producers and exporters of different food commodities, including soybeans, meat, cotton, grains, and ethanol. Projections suggest that Brazil is on track to become the world’s leading food exporter, given its large availability of arable lands (USDA, 2024; FAO, 2024a). However, this agricultural success has not translated into broad-based economic equality. Despite the rapid expansion of agribusiness, Brazil remains a country marked by significant income inequality and poverty. It is estimated that less than 1% of agricultural properties account for nearly half of the country’s rural land, while small properties, comprising 47% of all agricultural properties, occupy only 2.3% of the total rural area (FAO, 2021).

This concentration of land ownership and the dominance of monoculture systems have made Brazil one of the largest consumers of pesticides globally. According to FAO data, in 2021, Brazil applied 719.5 thousand tons of pesticides across its agricultural lands, a quantity equivalent to the combined consumption of the United States and China. Pesticide use per hectare in Brazil (10.9 kg/ha) exceeds that of the United States (2.85 kg/ha) and China (1.9 kg/ha), positioning Brazil as the world’s largest importer of pesticides, with 87% of its pesticide supply being imported (FAO, 2024b). Although data on pesticide use can vary slightly depending on the source, Brazil, United States, and China consistently rank among the top three countries in pesticide consumption and application.

Notably, it is estimated that 30% of the pesticides used in Brazil are currently banned in the European Union (Bombardi, 2019). Alongside this, the discrepancies between countries in terms of pesticide approval, application rates per hectare, and maximum residue limits (MRLs) in food and water are striking. For some pesticides, the MRLs allowed in Brazil can be up to 400 times higher than those in the EU, depending on the pesticide and crop in question (Bombardi, 2019). While this comparison focuses on Brazil and the EU, it highlights broader disparities between the Global North and South. Paradoxically, some of these banned agrochemicals can return to Northern countries through imported products such as coffee, orange juice, and soybeans, thereby exposing global populations to harmful pesticides that are otherwise prohibited. This occurs despite the presence of stringent policies aimed at evaluating agro-food products before they enter local markets (PAN, 2024). Additionally, the method of pesticide application plays a significant role in environmental contamination. For example, a method called aerial spraying, known for dispersing pesticides beyond the target area, has been banned in the EU since 2009 but remains legal in Brazil, although it has been debated in recent years (European Commission, 2009).

These deep differences regarding pesticide regulation between the Global North and South countries in the world contribute to a clear scenario of human and environmental health vulnerability in Latin America, Asia, and Africa. Addressing these disparities is urgent, and requires global initiatives, including investments in research to elucidate the most relevant biological risks associated with pesticide exposure, particularly regarding reproductive function, endocrine disruption, and carcinogenesis, special biological targets of these chemicals (Kim et al., 2017).

### 2.2 Initial efforts

Since 2001, the Brazilian Health Regulatory Agency (ANVISA), an autonomous institution of the Ministry of Health, has implemented the “RPRAF Program”- Reports on Pesticide Residue Analysis in Food. This initiative aims to dose pesticide residues in food samples collected from the different regions of Brazil using a multiresidue analysis approach. The aim of the program is to identify which pesticide residues are prevalent in the agro-food items, determining their concentrations, evaluating if these residues are in accordance with Brazilian regulations and, finally, mapping the most relevant pesticides for food safety in Brazil (ANVISA, 2019a).

The first consolidated report from this program dates back to 2002, in which there were only nine different food types evaluated. The 2008 report, the oldest available in full format, evaluated 17 different crops across the country, analyzing approximately 100 samples per crop. Across all crops, some samples contained pesticide residues not approved for that particular crop or residues exceeding the MRL. These violations ranged from 1% of samples for bananas and mangoes to 64% for bell peppers. The main conclusion of this early report was the confirmation of unauthorized pesticide use and, to a lesser extent, the presence of residues above the MRL (ANVISA, 2024b). From 2009 onwards, the program improved significantly in terms of data presentation, depth of discussion, and the number of crops and samples evaluated, seeking to cover all Brazilian states and a substantial proportion of the agro-food consumed in Brazil, reaching a remarkable of almost 12,051 food samples, 25 types of agro-foods, representing 70% of Brazilian consumption.

Unfortunately, in 2019, the program regressed and covered only 4,616 samples of 14 types of fruits and vegetables, representing about 30.9% of the agro-foods varieties consumed in Brazil. The program was further weakened in 2020 and 2021, ostensibly due to COVID-19 prevention measures, but it resumed in 2022 (Associação Brasileira de Saúde Coletiva, 2021). Despite its imperfections, the program represents a crucial milestone for Brazilian regulatory agencies. However, it is essential to critically assess the program’s results, as the classification of samples as satisfactory or unsatisfactory is based on MRLs established in Brazilian legislation, which can be hundreds of times higher than those in other countries (Panis et al., 2022). While the program has its limitations, it has been instrumental in revealing the chemical exposome associated with Brazilian agro-food, paving the way for studies focused on relevant pesticides, doses, routes, and exposure schedules.

### 2.3 Insights from pre-clinical studies

A substantial majority of pesticides have been identified as endocrine disruptors (Kim et al., 2017), which are agents capable of interfering with and disrupting various stages of the hormonal signaling pathway. These disruptions can occur from hormone synthesis, cellular secretion, and transport, to receptor binding, and eventual excretion (Kavlock et al., 1996). Given that the development and maintenance of male reproductive function are dependent on a delicate balance within the hormonal axis, particularly the hypothalamic–pituitary–gonadal axis, any disturbance in this homeostasis can lead to adverse effects on somatic testicular cells, germ cells, sperm maturation in the epididymis, and overall male reproductive function (Sharpe, 2020).

Furthermore, pesticides are known to impair male reproductive function through direct damage to cellular structures, such as DNA mutations and modifications in epigenetic signatures of the genome of the exposed individual (Moreira et al., 2021). A thorough understanding of these mechanisms is crucial for raising awareness about the health risks associated with pesticide exposure and for promoting more cautious and informed use of these chemicals, besides fostering tailored health strategies and supporting regulatory policies. In this context, experimental studies have an expressive contribution.

Recently, our laboratory conducted experimental studies based on the most prevalent agrochemical residues in Brazilian agro-food products, as reported by ANVISA in 2016. This ANVISA report analyzed 12,051 samples of 25 foods of plant origin collected between 2013 and 2015, representing the Brazilian population’s diet, investigating up to 232 different pesticides in the samples. In our study, prepubertal rats were exposed to acephate, carbendazim, and the dithiocarbamate mancozeb, based on the prevalence of these chemicals in the ANVISA report (Garcia et al., 2021; Aranha et al., 2021). These agrochemicals are often used in combination in several crops, especially fruit cultures, so their isolated and combined toxicity was investigated using a full factorial design to enhance the study’s accuracy. For male reproductive parameters, the comparison among groups was performed using the Generalized Linear Model (GLM) with a full factorial design. When an interaction among agrochemicals was detected, *a posteriori* test was conducted to investigate the nature of the interaction (Garcia et al., 2021).

Given that all the agrochemicals studied have been described as reproductive toxicants when administered individually (Wang et al., 2020; Liu et al., 2019; Elsharkawy et al., 2019), and exhibit some common toxicity mechanisms, it was hypothesized that exposure to these agrochemicals in combination would result in either additive (when they act together without amplifying or diminishing each other’s effects) or synergistic (where the effects exceed those expected from an additive interaction) reproductive effects. To test this hypothesis, the animals were distributed into eight experimental groups: receiving the vehicle, isolated pesticides, binary mixtures, or a ternary mixture. The doses administered were effective but did not induce systemic adverse effects, based on previous studies of the isolated pesticides’ toxicity (Garcia et al., 2021).

Contrary to expectations, our study provides evidence that these agrochemicals, when combined in binary or ternary mixtures, primarily interact in ways that result in antagonistic effects on the reproductive parameters assessed, *i.e.,* the interactions between agrochemicals in mixtures culminated in reduced impact on reproductive parameters, compared to the effects observed when each chemical is tested individually, as detailed below. All biometric parameters (e.g., body and organ weights) indicated an antagonistic effect of the binary and ternary combinations compared to the isolated agrochemicals; the presence of acephate or mancozeb, regardless of the experimental group, accelerated puberty onset. In addition, the evaluation of serum hormone levels and antioxidant enzyme activity in the testis showed that all pesticides combinations exhibited antagonistic effects on these variables as well as on histomorphometric parameters. These results indicated endocrine disruption effects of each isolated agrochemical on developing male rats, revealing patterns similar to those documented in adult animals. Despite the evident endocrine disruption caused by each individual agrochemical, the interactions observed in binary and ternary mixtures resulted in antagonistic effects, highlighting the complexity of predicting chemical interactions based solely on isolated exposures.

For liver biochemical and histopathological parameters, a factor analysis with Principal Component Analysis was employed. It was revealed that Glutathione S-transferase levels and histopathological findings presented positive correlations with binary mixtures containing acephate (AcCz and AcMz) and negative correlations with control and ternary mixture treatments. Moreover, acetylcholinesterase activity, liver/mass ratio, and certain histopathological findings were positively correlated with treatments involving binary mixtures with mancozeb (AcMz and CzMz). Once again, contrary to the initial hypothesis, the ternary mixture did not exacerbate adverse effects in the liver observed with isolated or binary combinations of agrochemicals. Instead, some adverse effects provoked by the exposures to isolated or binary combinations were not present in the ternary combinations, suggesting an antagonistic interaction (Aranha et al., 2021).

## 3 Future perspectives and conclusion

**FIGURE 1:**
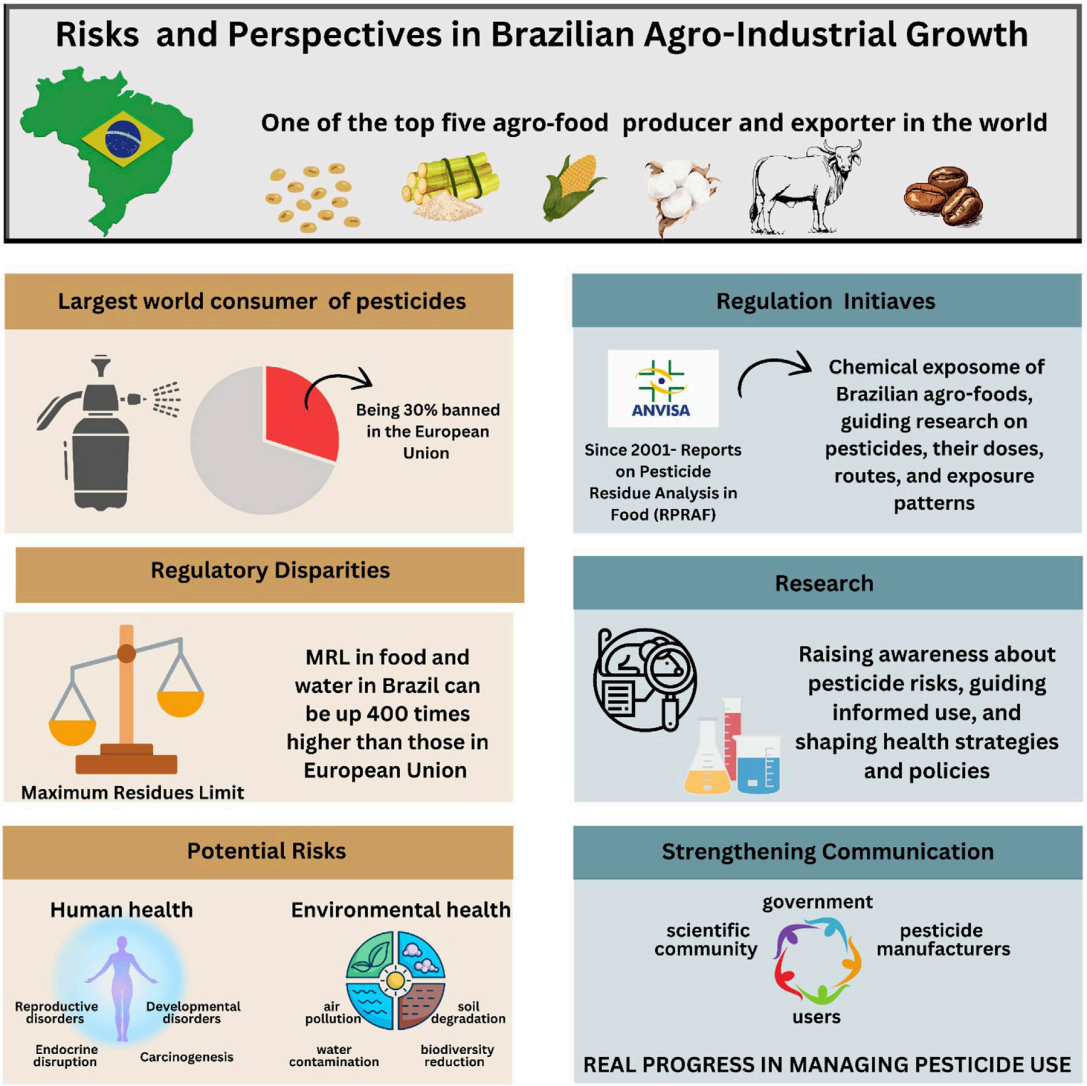
Risks and perspectives on the use of pesticides in the Brazilian agricultural scenario.

Figure 1 summarizes the risks and perspectives on the use of pesticides in the Brazilian agricultural scenario. Understanding the real risks associated with pesticide use is a significant challenge due to the diversity of chemical classes, their varying mechanisms of action, and the multitude of application methods and combinations used for different crops. Each of these variables influences the extent of toxicity and potential collateral damage to both human and environmental health. Additionally, the large amount of data available about this issue, from different sources including some of questionable credibility, often results in fragmented or biased interpretations based on incomplete segments of the overall picture. This fragmentation complicates an integrative assessment of pesticide-related issues and an effective problem-solving approach which encompasses environmental impacts as well as social, economic, and health perspectives.

To address these challenges, it is imperative to mobilize public opinion and, most critically, enacting supportive governmental policies. The scientific community plays a crucial role in this process by generating high-quality data that can inform policy decisions. Employing reliable and efficient methodologies is essential to meet the urgent demands of this issue. Experimental studies conducted under controlled conditions offer valuable insights, but it is also vital to apply advanced methods for complex data analysis and to evaluate conditions that closely mimic real-life exposure scenarios. This includes conducting exposome studies to assess interactions among key chemicals, measuring exposure levels that approximate environmental and occupational conditions, and simulating exposure schedules that reflect human ones, including chronic exposure to very low levels of pesticides residues.

Additionally, understanding individual biological variability is crucial for developing personalized strategies to mitigate adverse effects from previous exposures and to address current impacts of chemical exposure. Effective communication channels among the scientific community, government, pesticide manufacturers, and users are essential for achieving meaningful progress in managing pesticide use. Financial incentives for research should be prioritized, promoting advancements in understanding real risks and developing agricultural practices that adhere to sustainable principles.

## Data Availability

The original contributions presented in the study are included in the article/supplementary material, further inquiries can be directed to the corresponding author.
